# Rapid, Large-Scale Wastewater Surveillance and Automated Reporting System Enable Early Detection of Nearly 85% of COVID-19 Cases on a University Campus

**DOI:** 10.1128/mSystems.00793-21

**Published:** 2021-08-10

**Authors:** Smruthi Karthikeyan, Andrew Nguyen, Daniel McDonald, Yijian Zong, Nancy Ronquillo, Junting Ren, Jingjing Zou, Sawyer Farmer, Greg Humphrey, Diana Henderson, Tara Javidi, Karen Messer, Cheryl Anderson, Robert Schooley, Natasha K. Martin, Rob Knight

**Affiliations:** a Department of Pediatrics, School of Medicine, University of California, San Diego, La Jolla, California, USA; b Herbert Wertheim School of Public Health and Human Longevity Science, University of California, San Diego, La Jolla, California, USA; c Department of Urban Studies and Planning, University of California, San Diego, La Jolla, California, USA; d Halicioglu Data Science Institute, University of California, San Diego, La Jolla, California, USA; e Department of Electrical and Computer Engineering, University of California, San Diego, La Jolla, California, USA; f Campus Planning, University of California, San Diego, La Jolla, California, USA; g Division of Hypertension and Nephrology, School of Medicine, University of California, San Diego, La Jolla, California, USA; h Division of Infectious Diseases and Global Public Health, School of Medicine, University of California, San Diego, La Jolla, California, USA; i Department of Bioengineering, University of California, San Diego, La Jolla, California, USA; j Department of Computer Science & Engineering, University of California, San Diego, La Jolla, California, USA; k Center for Microbiome Innovation, University of California, San Diego, La Jolla, California, USA; Queen’s University Belfast

**Keywords:** COVID-19, SARS-CoV-2, wastewater epidemiology, high-throughput

## Abstract

Wastewater-based surveillance has gained prominence and come to the forefront as a leading indicator of forecasting COVID-19 (coronavirus disease 2019) infection dynamics owing to its cost-effectiveness and its ability to inform early public health interventions. A university campus could especially benefit from wastewater surveillance, as universities are characterized by largely asymptomatic populations and are potential hot spots for transmission that necessitate frequent diagnostic testing. In this study, we employed a large-scale GIS (geographic information systems)-enabled building-level wastewater monitoring system associated with the on-campus residences of 7,614 individuals. Sixty-eight automated wastewater samplers were deployed to monitor 239 campus buildings with a focus on residential buildings. Time-weighted composite samples were collected on a daily basis and analyzed on the same day. Sample processing was streamlined significantly through automation, reducing the turnaround time by 20-fold and exceeding the scale of similar surveillance programs by 10- to 100-fold, thereby overcoming one of the biggest bottlenecks in wastewater surveillance. An automated wastewater notification system was developed to alert residents to a positive wastewater sample associated with their residence and to encourage uptake of campus-provided asymptomatic testing at no charge. This system, integrated with the rest of the “Return to Learn” program at the University of California (UC) San Diego-led to the early diagnosis of nearly 85% of all COVID-19 cases on campus. COVID-19 testing rates increased by 1.9 to 13× following wastewater notifications. Our study shows the potential for a robust, efficient wastewater surveillance system to greatly reduce infection risk as college campuses and other high-risk environments reopen.

**IMPORTANCE** Wastewater-based epidemiology can be particularly valuable at university campuses where high-resolution spatial sampling in a well-controlled context could not only provide insight into what affects campus community as well as how those inferences can be extended to a broader city/county context. In the present study, a large-scale wastewater surveillance was successfully implemented on a large university campus enabling early detection of 85% of COVID-19 cases thereby averting potential outbreaks. The highly automated sample processing to reporting system enabled dramatic reduction in the turnaround time to 5 h (sample to result time) for 96 samples. Furthermore, miniaturization of the sample processing pipeline brought down the processing cost significantly ($13/sample). Taken together, these results show that such a system could greatly ameliorate long-term surveillance on such communities as they look to reopen.

**Author Video**: An author video summary of this article is available.

## INTRODUCTION

The recent SARS-CoV-2 (severe acute respiratory syndrome coronavirus 2) pandemic has revealed the imminent need for rapid surveillance at the community level to track potential outbreak clusters ahead of clinical diagnosis, particularly considering the important role of asymptomatic infection in transmission. Wastewater-based epidemiology has been extensively employed to monitor several enteric viruses such as poliovirus across several global settings (1, 2). Wastewater monitoring has additionally been used as a surrogate to track the extent and spread of SARS-CoV-2 in communities—particularly when diagnostic testing is limited. Previous work has shown the ability of wastewater-based surveillance to foreshadow trends in diagnosis and hospitalization rates ahead of clinical testing, thereby serving as a barometer of the community infection dynamics (3–5). By providing an indicator of disease burden/prevalence in a given community, wastewater monitoring can act as an early warning system; potential clusters can be advised to undergo diagnostic testing, increasing the opportunities for early public health interventions for individuals testing positive for SARS-CoV-2. Clinical diagnostics in tandem with wastewater-based surveillance systems can potentially provide a balanced community-wide perspective by increasing the chances of capturing both symptomatic and asymptomatic individuals. Wastewater-based surveillance can be particularly valuable at university campuses (which often self-maintain quasi-closed sewer systems) where the detection of virus in wastewater in buildings where one or more infected individuals are living or working can prompt testing of residents/employees in the affected buildings to diagnose and isolate individuals early on in their course of infection and avert outbreaks. With more than 700,000 cases (6) linked to university campuses in the United States alone, environmental or wastewater-based surveillance can alleviate the burden of frequent diagnostic testing by focusing testing effort on potential hot spots (7). Tracking SARS-CoV-2 signatures in sewage at the building level could enable targeted response strategies. Furthermore, high-resolution spatial sampling in a well-controlled context, coupled with detailed building use and occupancy data in a systematic manner integrated into a campus GIS (geographic information systems) network, can greatly augment data analytics (for instance, utilizing historical data to forecast high-risk locations). In addition to the overarching social consequence for robust and rapid identification of SARS-CoV-2 outbreaks on college campuses, the university setting can also serve as an ideal test for collating and relating information about SARS-CoV-2 incidence and spread, which can facilitate similar studies across different scales and different types of communities. Over the last few months, around 210 universities nationally and globally have implemented wastewater surveillance as a part of their SARS-CoV-2 monitoring programs (https://www.covid19wbec.org) (8–10). However, most of these campuses monitor either a few buildings or on an infrequent scale, and none monitor on a daily basis.

The University of California San Diego (UCSD) implemented a multifaceted adaptive approach as a part of its Return to Learn (RTL) program, which aimed to improve campus safety through three interdependent pillars: risk mitigation (e.g., masking, sanitation, and hygiene, campus dedensification, etc.), viral detection (e.g., asymptomatic and symptomatic testing, environmental monitoring, molecular sequencing), and public health intervention (e.g., isolation/quarantine, contact tracing, digital exposure notification). Environmental monitoring consisted of wastewater monitoring conducted in tandem with diagnostic testing and contact tracing. During the fall term of 2020, approximately 9,700 undergraduate and graduate students lived on the UCSD campus, and an estimated 4,000 employees worked on campus. All on-campus residents were also mandated to participate in the same free and campus-provided diagnostic COVID-19 (coronavirus disease 2019) tests on a biweekly basis (once every 2 weeks), which aided in the validation of the sensitivity and efficacy of the wastewater surveillance. Here, we report findings from our observational study of wastewater monitoring in these high-density buildings, and a cluster randomized study of manholes associated with residential buildings that were randomized to receive wastewater monitors at one of two time steps. The sensitivity of the monitoring enabled detection of a single asymptomatic individual in a building with 415 residents, thereby illustrating the potential for averting potential outbreaks on campus. Furthermore, quantitative analysis of the wastewater signatures from the isolation units helped glean insight into the microscale viral shedding dynamics and its potential implications on the relationship between the viral load in wastewater and positivity rates.

## RESULTS AND DISCUSSION

### Implementation of a large-scale wastewater surveillance program.

**FIG 1:**
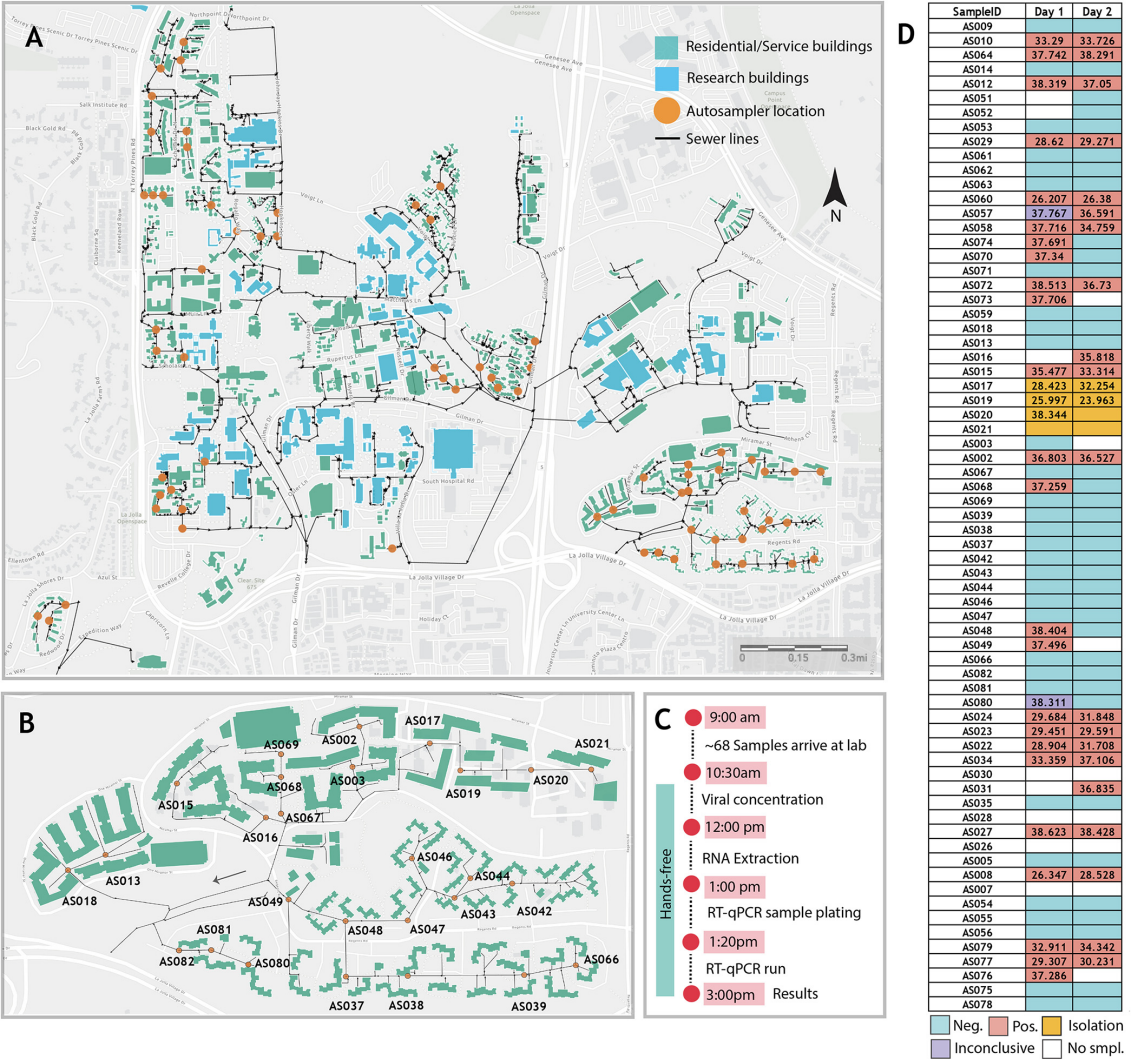
High-throughput wastewater surveillance scheme. (A) Map showing the locations of the 68 actively deployed autosamplers (denoted in orange) across the campus residences. (B) Snapshot of one of the residence clusters showing the locations of 27 samplers covering one of the zones with the highest occupancy on campus. All autosamplers have unique IDs which have been prelinked to the corresponding manholes on the GIS server. (C) Timeline of the daily wastewater sampling and analysis. (D) An example of the wastewater sample data reported over two consecutive days. The numbers in the cells indicate the measured cycle threshold values of the N1 gene for the respective sample. Amplification in at least two/three genes for both replicates was considered positive. The samplers indicated in yellow were collected from the isolation dorms on campus. Building-specific data have been deidentified in accordance with university reporting policies. Maps are the intellectual property of Esri and its licensors and are used under license. Copyright © 2021 Esri and its licensors. All rights reserved.

Analyses informed by the campus GIS were used to identify manholes or sewer cleanouts associated with each residential building on campus. Sixty-eight commercial autosamplers (that can collect time-weighted composite samples) were deployed across the campus residences. Autosamplers were first prioritized to the most proximal manhole to buildings with residential populations greater than 150. This decision was made based on agent-based network modeling of SARS-CoV-2 transmission on the UCSD campus, indicating that the highest risk areas for large outbreaks on campus were buildings containing the largest residential populations (11). In parallel to our observational study of wastewater monitoring in these high-density buildings, we additionally performed a cluster randomized study. Clusters of manholes associated with residential buildings were randomized to receive wastewater monitors at one of two time steps (end of November or end of December). The purpose of this ongoing cluster RCT (randomized crossover trial) was to evaluate the impact of wastewater monitoring on outbreak size in the associated buildings. In total, across our observational and cluster RCT studies, these 68 autosamplers covered 239 buildings, including the majority of the on-campus residence halls (Fig. 1A). One of the major bottlenecks in the implementation of a large-scale wastewater monitoring system on campus with daily sample collections is the sample processing time and labor. Here, we employed an automated, high-throughput wastewater processing pipeline which enables the processing of 96 wastewater samples in 4.5 h (Fig. 1C), reducing the required time, cost, and personnel dramatically.

### Data integration and visualization.

**FIG 2:**
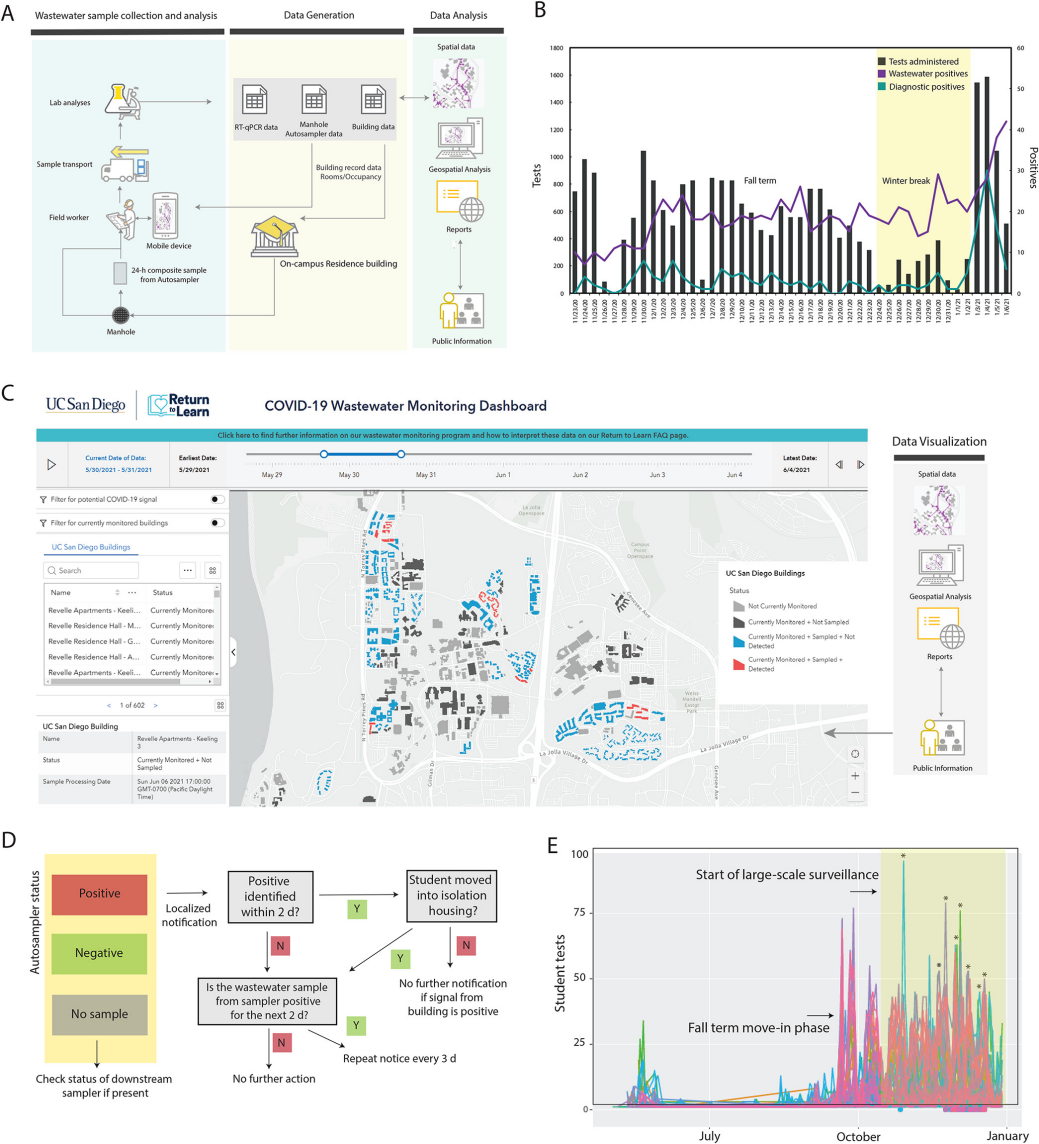
Wastewater surveillance workflow implementation. (A) Sample collection to analysis workflow. (C) Diagnostic testing and wastewater data shown for a 45-day duration. A spike in the wastewater and subsequently diagnostic testing was observed prior to the start of the winter term on 4 January 2021 (move-in began on 1 January 2021). (C) The interactive public wastewater monitoring dashboard showing the buildings monitored (black) and potential buildings that contributed to a positive wastewater signal (red). The dashboard is updated daily. A slider at the top of the dashboard enables the viewing of historic data. The public-facing dashboard can be accessed at https://returntolearn.ucsd.edu/dashboard/index.html. (D) Fall quarter 2020 notification process in the case of a positive signal detection from any of the autosamplers. Note that for buildings with public bathrooms, a campus-wide notice was sent. 2 d, 2 days.(E) Student testing rates associated with each wastewater sampler positive during the study period. The colors represent the individual manholes where the samplers were deployed at and recorded at least one positive result during the study period. The dots shown below the *x* axis indicate that a notification was sent to these students on the corresponding date. Selected testing peaks following wastewater notifications are indicated by asterisks on the plot (further details are provided in Table S2 in the supplemental material). Maps and dashboard are the intellectual property of Esri and its licensors and are used under license. Copyright © 2021 Esri and its licensors. All rights reserved.

Samples from the 68 wastewater samplers were collected daily by field staff. Each autosampler collected wastewater into a prelabeled sample bottle. Both the autosampler and the sample bottle are associated with a unique scannable barcode which were scanned by the on-site workers using a mobile app when samples were being picked up. The autosamplers were prelinked to campus asset codes for the manholes they are deployed at for ease of data integration. When pulling samples at particular sites, field staff can easily manage corresponding spatial features using their mobile phones and the samples can be tracked to the lab utilizing the unique identifier (ID). The samples were then brought to the laboratory for viral concentration and analyses. SARS-CoV-2 signatures in wastewater were elucidated via real-time quantitative PCR (RT-qPCR) screening for the three SARS-CoV-2-specific genes namely, N1, N2, and the E gene (Fig. 1D). The results for each sample were then pushed into the data reporting system, which can be viewed and analyzed by researchers (Fig. 2A). The obtained RT-qPCR results were then integrated with the campus GIS database in order to trace the potential sources of observed positive signals based on which buildings are upstream from an autosampler in the campus sewer network. For instance, if sampler B was positive but an upstream sampler A was negative, only the buildings contributing waste into the sewer between samplers A and B were assumed to be potentially associated with the positive wastewater signal. The spatially enabled sewer network and subsequent trace of samplers to buildings were stored in and performed by ArcGIS Pro 2.7 (Esri). These data were then visualized by leveraging the ArcGIS Online platform (Esri) into several maps and dashboards. A public-facing interactive wastewater map that displays buildings associated with positive samplers can be viewed at the bottom of the RTL dashboard at https://returntolearn.ucsd.edu/dashboard/index.html (Fig. 2C). Other visualizations include internal web maps that display the connection of sewer manholes, cleanouts, and buildings. This workflow increases efficiency and awareness concerning COVID-19 testing and tracing by integrating rich interconnected geospatial and lab results to accurately inform decision making. Targeted e-mail notices were sent to those residing or working in the specific building(s) which were deemed potential sources of the wastewater positive (Fig. 2D). Campus-wide notices were sent in the event a potentially positive building contained a common access area open to the public. The e-mails alerted individuals to the positive wastewater signal and encouraged individuals to obtain SARS-CoV-2 testing at no charge at any of the testing locations on campus or via the use of self-administered tests available at vending machines across the campus. Additionally, signs were placed in the interior hallways of affected buildings with information on the wastewater-positive status. Wastewater results were corroborated with the diagnostic test results among students living in campus buildings monitored by the wastewater program. During the fall 2020 term, students residing on or coming to campus were mandated to test every other week.

10.1128/mSystems.00793-21.2 TABLE S2 Testing uptake rates in buildings associated with each manhole pre- and postnotification after a wastewater-positive result. Download Table S2, DOCX file, 0.02 MB. Copyright © 2021 Karthikeyan et al. 2021 Karthikeyan et al. https://creativecommons.org/licenses/by/4.0/ This content is distributed under the terms of the Creative Commons Attribution 4.0 International license.

### Data analytics. (i) COVID-19 cases identified in relation to wastewater detection and notification.

From 23 November to 31 December 2020, a total of 1,574 wastewater samples were collected. Of the collected samples, a total of 692 were positive, 878 were negative, and 34 were inconclusive. Ninety-six of the total positives obtained were associated with isolation dorms, which served as positive controls. The proportion of samplers collected with a positive signal increased over time (Fig. 2B), consistent with increases in case numbers among residential students and the broader San Diego County over the same time period. Across this period, there were 59 cases diagnosed among on-campus students residing in buildings monitored by the wastewater program. Of these cases, 84.5% (*n* = 50) of these individual case diagnoses were preceded by positive wastewater samples (either in the days prior or the day of diagnostic testing), indicating that the wastewater program was highly sensitive in detecting cases. In only 8% of individual cases (*n* = 5) was the wastewater signal negative the days preceding individual diagnostic detection. Seven percent (*n* = 4) of individual cases were missed because no sample was obtained the day of or prior to diagnostic detection (see Table S1 in the supplemental material). Over the course of our surveillance, 23 cases were identified within 2 days after sending out a localized notice indicating the high overall response rate (Fig. 2E and Table S1). The identified students were moved to the designated isolation and quarantine buildings on campus, with the exception of select graduate students who can isolate in place (if living with family). One hundred on-campus cases were reported at UC San Diego during our wastewater surveillance (23 November to 31 December 2020). However, 85 new cases were recorded from 1 January to 7 January 2021, associated with students returning to their on-campus residence after winter break (compared to 11 unique cases associated with students who remained on campus) (Fig. 2B). During the same period, 435 cases were reported by students who were living off-campus in the San Diego area.

10.1128/mSystems.00793-21.1 TABLE S1 Categorization of COVID-19 cases in relation to wastewater sampling, detection, and notification occurring between 23 November 2020 and 31 December 2020. Download Table S1, DOCX file, 0.01 MB. Copyright © 2021 Karthikeyan et al. 2021 Karthikeyan et al. https://creativecommons.org/licenses/by/4.0/ This content is distributed under the terms of the Creative Commons Attribution 4.0 International license.

### (ii) Impact of wastewater-triggered notifications on testing uptake rates.

In order to study the impact of wastewater-related notifications and their impact on the testing rates, we analyzed the wastewater data from building(s) associated with individual manholes with notification and test data from those buildings. Data from 36 individual manholes which were associated with wastewater-positive results (hence targeted notifications) during the period of the study were examined. The study period also corresponded to the beginning of the large winter surge in COVID diagnoses in San Diego County and also with the holiday season. For each manhole, using the first notification date as the index date, the sum of the associated student test numbers over the 3 days prior to the first notification date was taken as the prenotification total test number, and the sum of the associated student test numbers on the first notification date plus the two subsequent days was taken as the postnotification total test number. From these pre- and postnotification total test numbers formed, two summary statistics were estimated: the difference in test numbers (postnotification minus prenotification) and the ratio of test numbers (postnotification divided by prenotification). Across the 36 manholes studied, the median ratio of postnotification test numbers to prenotification test numbers for the 36 manholes was 1.90 (bootstrap 95% confidence interval [CI], 1.48 to 3.17). The ratio for all but three manholes was over 1. The mean difference was 26.7 (normal theory 95% CI, 14.6 to 38.7). In almost all cases, the notification increased the testing rates 1.5× to 13× (Fig. 2E and Table S2). Because these data were obtained during a COVID surge, the positive post-prenotification difference in testing rates might reflect an increasing trend in COVID testing rates during the surge period. Therefore, we also conducted a permutation test to assess whether there was a statistically significant association between a notification event and increased COVID testing rates, controlling for any temporal background changes in testing intensity. With the randomly permuted dates, there is no association between notification and testing, by construction. On the other hand, any general increasing trend (due to notification) during these dates will still be captured in the permuted data. Because the permutation test *P* value is less than 0.001, we reject the null of no association between notification event and increasing in testing.

### (iii) Microscale viral shedding dynamics.

**FIG 3:**
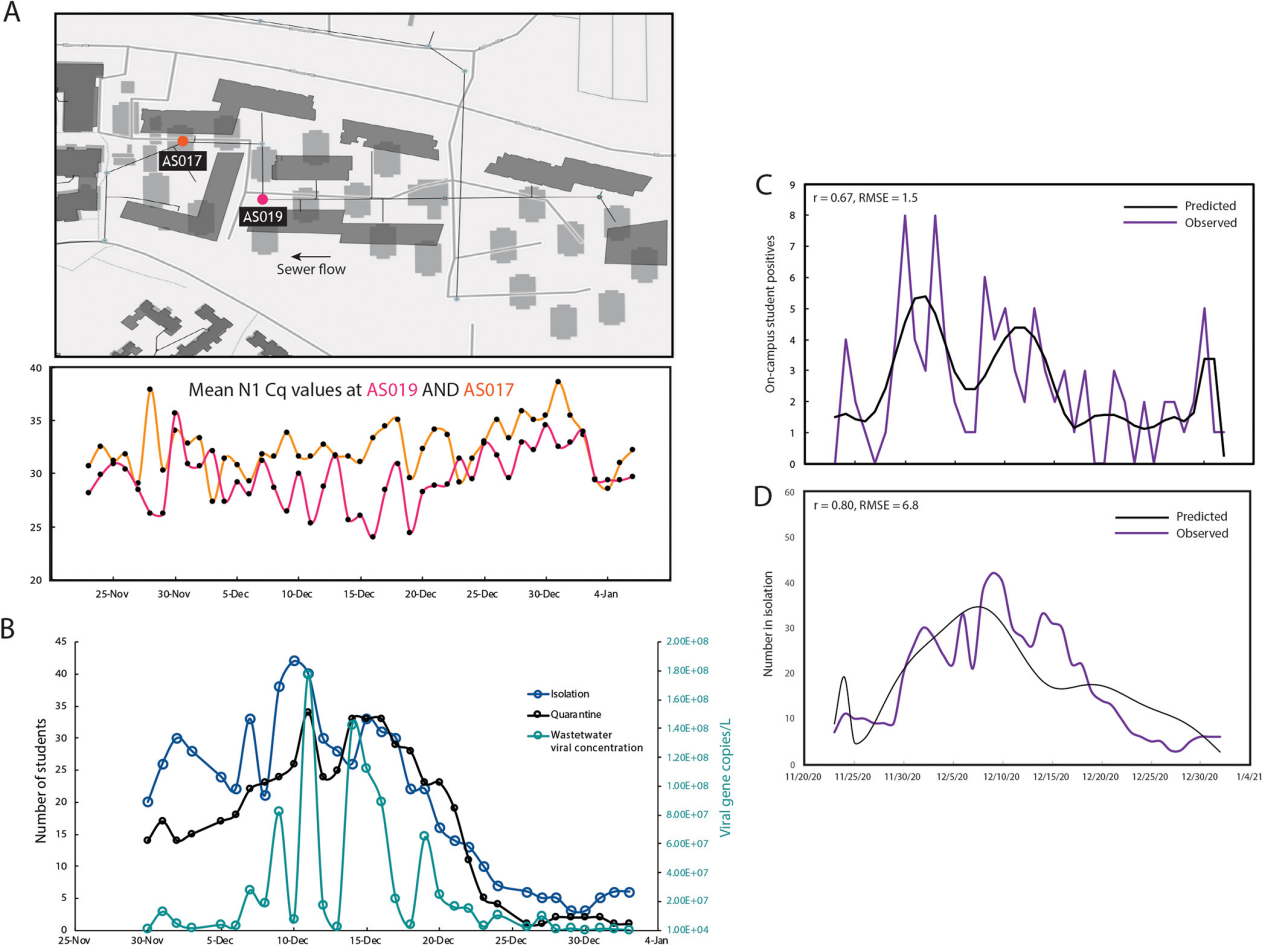
Quantitative interpretation of the wastewater data. (A) Snapshot of the sewer network showing the two autosamplers by isolation unit AS017 is downstream of sampler AS019 (associated with the isolation dorm). The mean *C_q_* values of the daily samples from the two samplers are shown in the bottom panel. (B) Mean viral gene copies per liter of sewage collected daily from the isolation dorms (sampler AS019) compared to the number of students in isolation/quarantine on the same day. (C) Measured daily caseload data compared to the predicted filter output with a 1-day sampling delay for all active on-campus samplers (mean, 0.67; root mean square error [RMSE], 1.5). (D). Measured daily caseload data compared to the predicted filter output with a 1-day sampling delay for the isolation unit sampler AS019 (mean 0.80, RMSE 6.8). Maps are the intellectual property of Esri and its licensors and are used under license. Copyright © 2021 Esri and its licensors. All rights reserved.

While SARS-CoV-2 signatures in wastewater can be valuable qualitatively, the granularity of its quantitative interpretation still remains to be elucidated, for example to determine the number of individuals in a building and their correlation to the SARS-CoV-2 signal in wastewater. Currently, the viral shedding dynamics and its effects on wastewater are still not well understood on a microscale which precludes the quantitative interpretation of such data (12). This is in part, due to the observed wastewater measurement’s dependence on individualized shedding patterns, where the shedding signature itself may be an indication of the onset or severity of infection. The SARS-CoV-2 signatures from isolation dorms were also being monitored on a daily basis in order to study individual viral shedding dynamics over time on a scale ranging from 1 to 100 infected persons per building. As a simple first step toward understanding these complex shedding dynamics, the aggregate of the collected wastewater signal from all campus samplers was used to find any correlations with the reported on-campus positive results. The positive caseload was modeled as the output to an infinite impulse response (IIR) filter with wastewater signal as an input. The resulting IIR filter (essentially the inverse of a filter capturing the shedding dynamics) was used, as it was found to be more meaningful to estimate the caseload. Furthermore, the campus sampler network lies on a gravity sewer, where a specific manhole sampling location will be affected by upstream nodes. Although the true shedding signal is expected to be diluted as it flows downstream, we found that a sampler (AS017) placed 276 ft downstream from the sampler at isolation building (AS019), exhibited a positive signal often correlating with the isolation building occupancy (Fig. 3A). Further study is required to quantify the persistence of a signal further downstream for any given sampling point. However, the persistence of a signal downstream combined with high probability of a signal erasure, due to the time and flow-weighted discrete wastewater measurements, at any given sampling point could indicate that the true shedding signal at a building is likely represented by a mixture of the collected wastewater signal from multiple associated samplers. Using these data, the transfer function of the filter was estimated using time-discrete data from 23 November 2020 to 1 January 2021 consisting of aggregate daily wastewater data from all campus samplers as the input and reported positive new cases as the output. Figure 3C shows the measured caseload data compared to the predicted filter output, with a 1-day sampling delay (since the samples are 24-h composites). Furthermore, the estimated filter was then applied to the data from the isolation building denoted by AS019, where the wastewater signal was consistently strong due to students in isolation. Over the course of the study, the number of students isolating in these dorms varied between 3 and 42 (Fig. 3B). Figure 3D shows the measured active caseload compared to the predicted filter output with a 1-day sampling delay. The estimated filter fit the isolation unit data with a correlation coefficient *r* of 0.80. These preliminary data highlight the importance of continuing to survey the isolation units in addition to campus-wide reporting for learning about the shedding dynamics on campus and shows that these may be scalable across campus.

### Conclusions and future perspectives.

Combining the wastewater and observational data can greatly aid in designing optimal intervention strategies using advanced statistical and epidemiological techniques, including time series analysis and agent-based modeling. Taken together, our data show wastewater-based epidemiology can be implemented successfully at the building level and serve as an important tool for early detection of COVID-19 outbreaks as well as being a cost-effective alternative to high-frequency diagnostic testing. Additionally, it could serve as a longer-term monitoring system when caseloads are lower and only testing a section of the population after encountering a wastewater-positive result (i.e., responsive testing instead of regular testing). The persistence of a signal in wastewater after a person is no longer infectious (due to extended viral shedding periods in stool) is currently one of the major challenges in wastewater surveillance. This can potentially obscure identification of more asymptomatic cases in the same building. SARS-CoV-2 viral genome sequencing of wastewater can also help resolve these by aiding in elucidating geospatial SARS-CoV-2 genotype distribution and is currently in progress. This could also help in early identification of outbreak clusters as well as in tracking newly emerging variants. The campus wastewater surveillance program has since expanded to cover more than 340 campus buildings, including the majority of nonresidential buildings, and 16,342 campus wastewater samples have been processed over the last 9 months.

## MATERIALS AND METHODS

### Site identification and autosampler deployment.

Sampling sites were identified via campus GIS, which provides mapping and flow direction of interconnected sewer lines and locations of manholes where samplers could be placed. Preliminary dynamic modeling indicated that the largest potential outbreaks would potentially occur within the largest residential buildings, so manholes associated with larger residential buildings were prioritized first.

Large-scale wastewater surveillance across campus began in November 202 with an initial 47 deployed samplers. This number was then increased to 68 by the end of 2020. Autosamplers (HACH AS950) were deployed across the identified sites. All the autosamplers were deployed at manholes and aboveground except for two which were deployed at sewer ejector pumps due to the inaccessibility of the associated manholes. The manhole covers were modified to enable the passage of the suction tube, thereby circumventing the need to install the sampler belowground and the need to open the manhole covers at every sample collection. All the autosamplers were retrofit with 1-liter Nalgene bottles (catalog no. 2104-0032; Thermo Fisher) in order to aid in easy and rapid sample retrieval. All autosamplers were programmed to retrieve samples at 1-h intervals over a 24-h period with a pre- and postpurge cycle. The tubing was disinfected after sample retrieval. The prebarcoded sample bottles were swapped daily. The autosampler barcode and the sample bottle barcodes were scanned by the field staff using the ArcGIS Survey123 mobile app (ESRI) which enabled automatic data integration into the ArcGIS Online environment for trace analysis.

### Sample analysis.

The SARS-CoV-2 viral RNA was concentrated from 10 ml of raw, unfiltered sewage samples and processed as described in our previous study (5). Detailed sampling to analysis protocol is also available at dx.doi.org/10.17504/protocols.io.bshvnb66.

Briefly, 10 ml of raw sewage was concentrated using an automated affinity capture magnetic hydrogel particle (Ceres Nanosciences Inc., USA)-based concentration method using a KingFisher Flex liquid-handling robot platform (Thermo Fisher Scientific, USA) (13). The concentrated viral RNA was then extracted using the MagMAX Microbiome Ultra Nucleic Acid isolation kit (catalog no. A24357 and A24358; Applied Biosystems) using 96-well plates. The RNA was eluted in 50-μl nuclease-free water and used for SARS-CoV-2 real-time RT-qPCR. The RT-qPCRs were carried out in a CFX384 real-time system (Bio-Rad) thermocycler in 384-well plates for three gene targets (N1, N2, and E gene). qPCR sample plating was performed using an EpMotion automated liquid handler (Eppendorf, Germany) in 384-well plates. To detect inhibition specific to sewage samples, the positive-control RNA ladder was run with every qPCR run (5 1:10 serial dilutions of a positive control). If quantification cycle (*C_q_*) values for the no dilution to 1:100,000 dilution were not significantly different, then PCR inhibition in the RNA extracts was considered unlikely. Two-tailed *t* tests at a 95% confidence interval were used to determine if the average *C_q_* values are statistically significant from the spiked-in water control. In addition, an internal amplification control (IAC) was included in every run to control for PCR inhibition. Viral ladder, positive controls, and extraction blanks were included in every run. A synthetic RNA encoding the E and N genes of SARS-CoV-2 was used as the positive control (catalog no. CS317402; Promega Corp.). A ladder of sixfold dilutions was run with every qPCR run. The no-template control (NTC) was nuclease-free water Amplification of two/three genes was regarded as positive, while one/three was regarded as inconclusive (only *C_q_* values of <40 in all targets were considered positive). If samples were inconclusive, an aliquot of the stored sample was processed and run again to confirm, and data were interpreted accordingly. Pepper mild mottle virus (PMMoV) was also screened to adjust for daily load changes. The *C_q_* values for the N1 gene ranged from 21.121 to 38.513 for the wastewater samples with an average of 33.198 for the samples measured during the study. The average *C_q_* values for the isolation dorms were 29.067 (standard deviation [SD], 2.52). Recovery experiments were conducted by spiking in serial dilutions of heat-inactivated SARS-CoV-2 viral particles into raw sewage samples. All RT-qPCR samples were run in replicate. Due to time sensitivity and role in campus decision-making, the wastewater data from the residences were primarily used qualitatively for decision-making (however, quantitative analyses were also performed for research purposes).

### Automated data reporting.

The wastewater data reporting is automated by AUM (auto-update microservice), a microservice hosted by AWS Lambda, through a docker image for all the dependencies, such as Google Sheet API. Each day when the raw *C_q_* values are uploaded, the microservice automatically converts the *C_q_* values to a format that can be cross referenced with the daily plate-map and automatically update the data report on a Google Sheet that has the longitudinal wastewater data across all deployed samplers. The data from the sheet are then linked to the GIS server to automatically update the dashboard, thereby streamlining the data integration process. The detailed architecture is available at the Github repository (https://github.com/CrisZong/AUM.git) for replication and deployment.

### Ethics.

This project was discussed with our institutional review board, and the wastewater component was not deemed to be human subject research, as it did not record personally identifiable information. In particular, although the collected data could potentially identify a building in which a resident had active COVID, university-mandated surveillance testing and notification protocols also identified such buildings. When such locations were identified, we notified individuals in these locations about the wastewater finding and recommended increased testing, but as discussed above, the testing itself did not identify specific individuals. While the wastewater testing component may have contributed to more timely notification, it did not contribute additional data elements which could compromise privacy. However, given the high individual variation of the human microbiome and the fact that human DNA can be isolated from stool samples, it is appropriate that wastewater surveillance projects such as this one should be reviewed as potential human subject research.
