# Supplementary material for: Rapid, Large-Scale Wastewater Surveillance and Automated Reporting System Enable Early Detection of Nearly 85% of COVID-19 Cases on a University Campus
Source: mSystems. 2021 Aug 10;6(4):e00793-21. doi: 10.1128/mSystems.00793-21 (PMC8409724; doi:10.1128/mSystems.00793-21)
Supplement: TABLE S1 [file msystems.00793-21-st001.docx]

**Supplemental Table S1:** **Categorization of COVID-19 cases in relation to wastewater sampling, detection, and notification occurring between November 23, 2020 and December 31, 2020.**

| Diagnoses of COVID-19 cases in relation to wastewater detection and notification | | **n** | **%** |
| --- | --- | --- | --- |
| Positive signal preceding diagnosis | Case diagnosed **less than 3 days** from a notification | 23 | 39% |
|  | Case diagnosed **more than 3 days** from a notification | 7 | 12% |
|  | Case diagnosis coincident with positive signal (no notification) | 5 | 8% |
|  | Detected but downstream from isolation building (no notification) | 4 | 6% |
|  | Detected but notification occurred the day after case diagnosed because of notification delay | 5 | 8% |
|  | Case diagnosed where there was a known isolation (no notification) | 6 | 10% |
|  | Case diagnosed but not detected by wastewater | 5 | 8% |
|  | Sample not collected and no notification | 4 | 7% |
|  | **Total cases diagnosed** | **59** | **100%** |
